# Evaluating global health initiatives to improve health equity

**DOI:** 10.2471/BLT.23.290531

**Published:** 2023-10-31

**Authors:** Shams El Arifeen, John Grove, Peter M Hansen, James R Hargreaves, Hope L Johnson, Mira Johri, Esther Saville

**Affiliations:** a Maternal and Child Health Division, International Centre for Diarrhoeal Disease Research, Bangladesh.; b Office of the Executive Director, The Global Fund to Fight AIDS, Tuberculosis and Malaria, Geneva, Switzerland.; c Global Financing Facility for Women, Children and Adolescents, Washington, DC, United States of America.; d London School of Hygiene and Tropical Medicine, London, England.; e Gavi, the Vaccine Alliance, Geneva, Switzerland.; f Département de gestion, d’évaluation et de politique de santé, École de santé publique de l’Université de Montréal, 7101, avenue du Parc, Montréal, Québec H3N 1X9, Canada.

Global health initiatives are multistakeholder partnerships that mobilize and disburse resources to address global health challenges, often by supporting implementation of health programmes in low-and middle-income countries.^1^ These initiatives have made enormous contributions to saving lives and improving health globally, and are vital to the realization of sustainable development goal (SDG) 3 to ensure healthy lives and promote well-being for all at all ages.^1^^,^^2^ However, some members of the global health community have criticized the ways these initiatives work, notably in relation to power imbalances between donor and implementing partners in priority-setting and decision-making.^1^ These imbalances can translate into questions of whose knowledge, vision and voice drive organizational direction.

We are writing as representatives of the evaluation units and evaluation advisory bodies of three prominent global health initiatives, to reflect on challenges and solutions to strengthening health equity via improved evaluation. As part of its mission to save lives and protect people’s health by increasing equitable and sustainable use of vaccines, Gavi, the Vaccine Alliance, helps vaccinate almost half the world’s children against deadly and debilitating infectious diseases.^3^ To ensure that all women, children and adolescents can survive and thrive, the Global Financing Facility for Women, Children and Adolescents, a multistakeholder global partnership housed at the World Bank, supports 36 low- and lower-middle-income countries with financing and technical assistance to develop and implement prioritized national health plans to scale up access to affordable, quality care.^4^ The Global Fund, a worldwide partnership to defeat human immunodeficiency virus, tuberculosis and malaria, and ensure a healthier, safer, more equitable future for all, works to fight the deadliest infectious diseases, challenge the injustices that fuel them and strengthen health systems in more than 100 countries.^5^ In 2019, our organizations collectively raised and invested in excess of 6 billion United States dollars (US$), representing approximately 14% of all development assistance for health.^6^ In 2021, to strengthen the global response to the coronavirus disease 2019 (COVID-19) pandemic, donors entrusted our organizations with more than US$ 14 billion, representing roughly 21% of all development assistance for health.^6^

A core strategic focus for the global initiatives for health has been to improve access to essential vaccines, medicines and technologies for priority conditions.^1^ The goal of *Transforming our world: the 2030 agenda for sustainable development* drives us also to advance through transformative policies with the potential to reshape underlying socioeconomic and political structures.^7^ Here we discuss how reshaping organizational evaluation processes can enable us to deliver better on our mandates and on the SDGs.

## The importance of evaluation

To address key learning needs, major global health initiatives often invest in various forms of evidence generation and use, including independent evaluation. Global health evaluation is designed to deliver essential global public goods by producing knowledge on what works for development. For these initiatives, evaluation can offer vital evidence to accelerate the introduction and scale-up of innovations, to optimize the scientific and technical quality of investments and to assess delivery of results. Evaluation should thereby equip global health initiatives for course-correction and transformation. Evaluation also aspires to build mutual accountability that strengthens governance of these initiatives within a multilateral system, contributing to effective multistakeholder partnerships and good global governance, that is, to contribute to SDG 17: strengthen the means of implementation and revitalize the global partnership for sustainable development (target 17.16).^8^ Exemplifying this partnership model, for each of our three global health initiatives, the evaluation unit is based within the Secretariat, while the evaluation advisory body is independent of the Secretariat and reports to the Board.

## What are the equity issues?

Often, stakeholders from high-income countries play a leading role in setting and executing global health initiatives' organizational strategies.^1^ In the evaluation sphere, this power imbalance with the intended beneficiaries of the initiatives’ programmes can manifest in three ways. First, due to asymmetries of voice and participation, decisions that affect what questions are addressed by evaluation, when and how evaluations are undertaken, and how their results are used, are often made in high-income countries, despite being about programmes and people in low- and middle-income countries. Second, due to factors such as entry barriers and specific criteria of excellence and expertise, a relatively small number of actors currently conducts the independent evaluations commissioned by our organizations. Those awarded contracts are often specialized consultancy firms and individual consultants with notable experience and abilities; however, repeated recourse to the same firms limits the range of viewpoints and methodological skills applied in evaluations, and may negatively influence the quality of recommendations. Moreover, a concentrated market structure poses risks to independence, as firms may be less critical of organizations if they serve frequently as repeat service providers. Third, for centralized evaluations of high strategic importance, current evaluators are overwhelmingly from high-income countries, while programmes and activities being evaluated are in low- and middle-income countries. In addition to reinforcing pre-existing power differentials, this situation may compromise evaluation quality and credibility, as external evaluators may in some instances lack the cultural, historical and linguistic knowledge required for deep understanding of programme successes, failures and options for improvement, and may have less local traction to secure interviews and access data. Reliance on external partners from high-income countries can also hinder uptake and use of evaluation findings at country level and undermine country leadership.

We have witnessed some positive shifts in recent years, as global health initiatives and other development partners have strengthened their connections with in-country research and technical institutions. However, more far-reaching efforts are required to achieve equity.

## Proposed way forward

Recognizing that evaluation contributes to decision-making in our organizations, and that the opportunity to lead the design and conduct of evaluations builds capacity, careers and sustainability, we are joining forces to ensure enhanced representation and voice for low-income countries. We are committed to promoting equitable partnership models in all aspects of how our global health initiatives use and generate evidence, for internal and external learning, by shifting power dynamics and strengthening the central role of in-country research and technical institutions.

As an initial step, we aim to facilitate greater learning led by and for actors in low-and middle-income countries, especially in programme-recipient countries, and greater mutual learning among stakeholders from donor and recipient countries in the independent evaluations we commission. We will initiate four coordinated actions. First, identify and address barriers. We will work together, within and across our organizations, to analyse the barriers and bottlenecks that impede qualified candidates from recipient countries from applying, advancing or being awarded tenders. Barriers to application may include factors such as lack of information on requests for proposals, short time frames, linguistic factors, difficult administrative processes and high-risk up-front costs, among others. Factors that hinder meritorious candidates from advancing in application processes may include specific notions of competencies and excellence reflected in technical selection criteria and used by tender review committees. Second, vision setting. We will conduct a landscape analysis of successful approaches to strengthening partnerships between global health initiatives and local research and technical institutions, to learn what works, develop an evidence-informed common vision and align efforts around shared priorities. Third, shifting operations. Based on these learnings, we will work together within and across organizations to shift operations. Modifications to approaches to engaging evaluation partners are envisaged, including, where relevant, changes to tender and procurement processes. Strengthening engagement of evaluation units within health ministries or other relevant government agencies may also reinforce country leadership of the evaluation agenda within a given country context. Fourth, strengthening partnerships with and cross-learning among local research and technical institutions. Working together, we aim to expand and diversify the array of highly qualified evaluators able to guide our organizations. A first step, already initiated, is to tap more effectively into the pool of qualified consultants from countries benefitting from the support of the global health initiatives, consultants from low- and middle-income countries, consultancy firms, research institutes and think tanks. Furthermore, recognizing that evaluation should make a positive contribution to local empowerment and realization of the SDGs,^9^ we envisage joining forces to nurture early-stage talent. Mechanisms may include matching and pairing new and experienced evaluators to offer mentorship; supporting capacity-building partnerships with in-country research and technical institutions; facilitating learning opportunities, joint projects and placements for students and early-career evaluation specialists; and investing in technologies and processes that facilitate two-way dialogue.

We believe that strengthened evaluation processes within global health initiatives will be critical for improving long-term development outcomes, enabling our institutions to be more effective, credible, accountable and legitimate.^10^ Empowering those who are close to the ultimate programme beneficiaries to shape evaluations will enable us to better deliver on our missions by speeding progress towards more context-informed, effective strategies. This change of practice will also strengthen our ability to address equity issues and to inform actions and investments to address disparities, with strengthened learning and accountability to help achieve a more equitable future.
